# Creation of a Low-Alcohol-Production Yeast by a Mutated *SPT15* Transcription Regulator Triggers Transcriptional and Metabolic Changes During Wine Fermentation

**DOI:** 10.3389/fmicb.2020.597828

**Published:** 2020-12-14

**Authors:** Qing Du, Yanlin Liu, Yuyang Song, Yi Qin

**Affiliations:** ^1^College of Enology, Northwest A&F University, Yangling, China; ^2^Shaanxi Engineering Research Center for Viti-Viniculture, Yangling, China; ^3^National Forestry and Grassland Administration Engineering Research Center for Viti-Viniculture, Yangling, China

**Keywords:** low-alcohol, *SPT15*, RNA-seq, metabolome, wine, *Saccharomyces cerevisiae*

## Abstract

There is significant interest in the wine industry to develop methods to reduce the ethanol content of wine. Here the global transcription machinery engineering (gTME) technology was used to engineer a yeast strain with decreased ethanol yield, based on the mutation of the *SPT15* gene. We created a strain of *Saccharomyces cerevisiae* (YS59-409), which possessed ethanol yield reduced by 34.9%; this was accompanied by the increase in CO_2_, biomass, and glycerol formation. Five mutation sites were identified in the mutated *SPT15* gene of YS59-409. RNA-Seq and metabolome analysis of YS59-409 were conducted compared with control strain, suggesting that ribosome biogenesis, nucleotide metabolism, glycolysis flux, Crabtree effect, NAD^+^/NADH homeostasis and energy metabolism might be regulated by the mutagenesis of *SPT15* gene. Furthermore, two genes related to energy metabolism, *RGI1* and *RGI2*, were found to be associated with the weakened ethanol production capacity, although the precise mechanisms involved need to be further elucidated. This study highlighted the importance of applying gTME technology when attempting to reduce ethanol production by yeast, possibly reprogramming yeast’s metabolism at the global level.

## Introduction

Over recent decades, the alcohol concentration of wines produced by many warm regions around the world has increased by approximately 2% (v/v) (Goold et al., 2017). This is mainly due to the increasing preference of consumers for well-structured, full-bodied, and ripe-fruit wines and those wines are generally made from more mature grapes. Also, this has been exacerbated by global warming, which leads to higher sugar content in the grape varieties used to make wine (Varela et al., 2015; Rolle et al., 2017). Prompted by various reasons regarding wine quality, economic variables, and health concerns caused by high levels of alcohol, strategies aimed at reducing ethanol concentrations without impairing wine organoleptic quality have been performed in different ways (Varela et al., 2015; Dequin et al., 2017). Generally, microbiological strategies relating to the isolation and/or generation of the yeast strains used to make wine have proved to be the simplest and most economical methods, including *Saccharomyces cerevisiae* and non-conventional yeast species (Tilloy et al., 2015; Varela and Varela, 2018).

Engineering yeast strains with the capacity of redirecting carbon away from ethanol production to other endpoints is an effective approach and thus far, enhancing glycerol production has testified to be the most effective method (Otterstedt et al., 2004; Varela et al., 2012; Tan et al., 2016). Classical gene modification (GM) technologies have achieved increasing glycerol formation to reduce ethanol production by the manipulation of single or several genes. However, high concentrations of by-products, such as acetate, acetaldehyde, and acetoin, were generated in these previous experiments (Cambon et al., 2006; Varela et al., 2012). Since these by-products could have a significant negative effect on the flavor of wine, concerted efforts have been made to reduce the formation of these by-products (Cambon et al., 2006; Ehsani et al., 2009). Additionally, a combination of adaptive evolution and breeding strategies is applied to develop a low-alcohol yeast for wine making with higher levels of glycerol production; this strain reduced ethanol production by 1.3% (v/v) without the formation of undesirable by-products (Tilloy et al., 2014). Other researchers have focused on non-*Saccharomyces* strains in an attempt to reduce the ethanol content of wine; this is because such strains are known to exhibit different respiro-fermentative regulatory mechanisms when compared to *S. cerevisiae* (Quiros et al., 2014). Nevertheless, those non-*Saccharomyces* strains generally possess a weak capacity to complete wine fermentation on their own and must be accompanied by *S. cerevisiae* (Hranilovic et al., 2020). Consequently, there is significant interest in developing more effective strategies to balance low-ethanol wine production efficiency with good organoleptic qualities.

In this study, an alternative approach, global transcriptional machinery engineering (gTME), was used to develop strains of *S. cerevisiae* with reduced ethanol-production ability. The gTME technology was carried out by mutating the general transcription factor Spt15p, the TATA-binding protein (Alper et al., 2006); this protein plays a key role in the action of RNA polymerase and is one of the main DNA binding proteins that regulate promoter specificity in yeast (Eisenmann et al., 1989). The gTME technology is first used to improve the glucose/ethanol tolerance of *S. cerevisiae*, which shows the ability to re-program global gene transcription and change the complex phenotype of yeast strains (Alper et al., 2006; Alper and Stephanopoulos, 2007). Since then, several research studies have used the gTME approach to optimize the ethanol tolerance and ethanol production capacity of *S. cerevisiae*, and successfully demonstrated that gTME is advantageous when attempting to regulate the ethanol metabolism of yeast strains (Yang et al., 2011; Seong et al., 2017). In the present study, we used gTME technology to weaken the capacity of yeast to produce ethanol and ultimately created a strain of *S. cerevisiae* (YS59-409) with a low yield of ethanol production. RNA-Seq and metabolomic analysis were also conducted in an attempt to understand the metabolic mechanisms underlying the modified phenotype of YS59-409. This study highlighted the critical role of the *SPT15* regulator in reducing ethanol production in yeast and provided comprehensive insights to understand the molecular mechanisms of a new low-ethanol yeast.

## Materials and Methods

### Plasmids, Strains, *SPT15* Mutant Library Construction and Culture Conditions

We used *S. cerevisiae* YS59 (MATα; ura3-52, leu2-3, and his 5-519) (Liu et al., 2007) as the host strain and then amplify the open reading frame of *SPT15* gene from genomic DNA of YS59. The *SPT15* gene was inserted into the restriction sites between *Bam*HI and *Eco*RI using the pY16 vector, which was flanked with *TEF1* promoter and *CYC1* terminator (pY16-SPT15); the plasmid has a *URA3* selective marker, an ampicillin resistant marker and a *CEN/ARS* element (low copy).

The yeast mutant library was created by random mutagenesis (error-prone PCR) of the *SPT15* gene. Firstly, the *SPT15* mutant library was generated using the Diversify^TM^ PCR Random Mutagenesis kit (Clontech) with pY16-SPT15 as template. Plasmids obtained were transformed into *Escherichia coli* JM109 to produce a primary library for *SPT15* mutants. From the sequencing of 20 randomly selected colonies, mutations were found at 2–10 sites without preference. Then library plasmids were transformed into *S. cerevisiae* YS59 and incubated at 25°C on solid SD to generate a yeast library for *SPT15* mutant.

The plasmid was transformed into yeast cells by the lithium acetate method (Gietz and Woods, 2001). The strains and plasmids used in this procedure are summarized in Supplementary Table 1. *S. cerevisiae* strains were pre-cultured overnight in YPD medium (1% yeast extract, 2% peptone, and 2% glucose) at 30°C for non-selective propagation. The selective culture of engineered strains was conducted in SD medium (0.67% YNB, 0.077% Ura DO Supplement, and 2% glucose) at 30°C.

### Alcoholic Fermentation

Alcoholic fermentation was carried out using an inoculum of 5 × 10^5^ cells/mL in a shaking incubator at 25°C at 150 rpm; all cultures were carried out in triplicate. The medium was similar to Triple M medium as reported previously (Spiropoulos et al., 2000), and consisted of 75 g/L glucose, 75 g/L fructose, 6 g/L tartaric acid, 3 g/L malic acid, 0.5 g/L citric acid, 1.7 g/L yeast nitrogen base without amino acids, 1 g/L ammonium phosphate, 2 g/L casamino acids, 0.8 g/L L-arginine,1 g/L L-proline, 0.1 g/L Tryptophan, and 4 mL ergo stock (composed of 250 mL/L Tween80, 750 mL/L 95% ethanol and 2.5 g/L ergosterol), with pH 3.25.

### Site-Directed Mutagenesis of the *SPT15* Gene and Fermentation Assays for Recombinant Strains

Mutations in *SPT15* in strain YS59-409 were identified by DNA sequencing. For site-directed mutagenesis, plasmid pY16-SPT15 (containing the non-mutated *SPT15* gene) was used as a template with primers designed to the target nucleotide substitutions (Supplementary Table 2); these reactions were carried out with a Mut Express II Fast Mutagenesis Kit (Vazyme, China) in accordance with the manufacturer’s directions. The reconstructed plasmids were then transformed into *S. cerevisiae* YS59 in order to obtain recombinant strains (Supplementary Table 1). The effects of mutation on the ethanol production capacity of strains were performed in Triple M media.

### RNA-Seq Analysis

RNA-Seq analysis was used to investigate the transcriptional differences between the low-ethanol-production strain YS59-409 and the control strain YS59-pY16. Three independent samples were collected from the mid-log phase of fermentation for RNA extraction. Total RNA was extracted using the procedures described previously (Li et al., 2019). Agarose gel (1%) electrophoresis and a spectrophotometer (NanoDrop ND1000, United States) were then used to detect the purity and concentration of samples. Bioanalyzer (Agilent 2100, United States) was used to detect the RNA integrity numbers (>8.0) of these samples to satisfy the particular requirements of RNA-seq. The cDNA library construction and RNA sequencing were carried out by Beijing Genomics Institute (Shenzhen, China) using standard protocols. The RNA-Seq data generated in this study were submitted to NCBI Sequence Read Archive (SRA) under the accession number PRJNA548495.

To compare the transcriptomes of the mutant and control yeast strains, we used Bowtie2 to map clean reads to the reference gene and then used HISAT to reference the genome of *S. cerevisiae* S288c. Gene expression levels were quantified using the FPKM method (Trapnell et al., 2012). Screening of differentially expressed genes (DEGs) between the two strains was conducted using the NOISeq method (Tarazona et al., 2011) based on a foldchange (log_2_ Ratio) ≥1.5 and a divergent probability ≥0.8. Functional enrichment analysis of Gene Ontology (GO) and KEGG pathway enrichment analysis of DEGs were conducted by comparison with the entire genome background (Bonferroni-corrected *P*-value < 0.05).

### Metabolomics Analysis

The method used to prepare samples (six independent replicates) from the low-ethanol-production strain for metabolomic analysis was consistent with that used for RNA-seq analysis. Metabolites were extracted by vortex blending approximately 1 g of cells (fresh weight) in 1 mL of cold methyl alcohol for 30 s. Cells were then lysed by ultrasonication for 15 min and centrifuged for 15 min at 12,000 × *g* at 4°C; an aliquot of 200 μL of the supernatant was used for further analysis. Samples were analyzed by Shanghai Biocluster Biotech Co., Ltd. (China) using the Ultimate 3000 LC system coupled with an Orbitrap Elite mass spectrometer (Thermo, United States). The separation was performed on a Hypergod C18 column (100 mm × 4.6 mm 3 μm) at 40°C. A flow rate of 0.3 mL/min, and an injection volume of 4 μL, were used in all analyses; this was followed by auto-sampling at 4°C. Mass spectrometry detection used negative polarity with the following parameters: heater temperature, 300°C; sheath gas flow rate, 45 arb; auxiliary gas flow rate, 15 arb; sweep gas flow rate, 1 arb; spray voltage, 3.2 kV; capillary temperature, 350°C, and an S-Lens RF Level of 60%.

With regards to multivariate statistical analysis, principal component analysis (PCA) and orthogonal partial least squares discriminant analysis (OPLS-DA) were performed using SIMCA-P version 13.0 software (Umetrics AB, Sweden) to separate the two groups of data. We then searched for differential metabolites using the variable importance in the projection (VIP) value of the OPLS-DA model (VIP > 1) in combination with the *p*-value of the *t*-test (*p* < 0.05). Qualitative metabolites were characterized by searching an online database^1^. Metabolic pathway analysis was carried out using MetaboAnalyst 3.0^2^.

### Analytical Methods

We measured the weight loss in CO_2_ from each sample by weighing the fermenters on a daily basis. Cell growth was recorded by a microplate reader at OD_600 nm_ (BioTek, ELx800, United States). Ethanol yields were analyzed using an SBA-40C biological sensor analyzer (Biology Institute of Shandong Academy of Sciences, China). The content of reducing sugar in each sample was measured by the DNS method (Hu et al., 2008) with glucose as a standard using an ultra-violet spectrophotometer. Concentrations of glycerol and acetic acid were determined with an Enology Analyzer Y15 (BioSystems, Spain).

### Data Analysis

Statistically significant differences between the wild-type (YS59-pY16) and mutant yeast strains (YS59-409) were determined using the Student’s *t*-test. The effect of site-directed mutagenesis and gene knockout on the fermentation characteristics of the two strains were further determined by one-way analysis of variance (ANOVA) and Duncan’s test. The confidence level for both tests was 95% and all analyses were carried out using SPSS version 19.0 software (SPSS Inc., United States).

## Results and Discussion

### Selection and the Characteristics of the Low-Ethanol-Yield Strain

**FIGURE 1:**
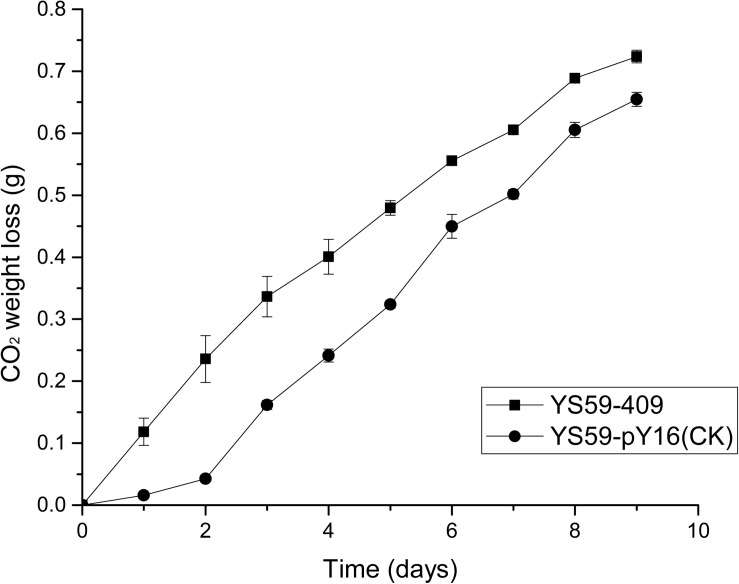
CO_2_ weight loss (g). Black square represents *S. cerevisiae* YS59-409 and black round represents *S. cerevisiae* YS59-pY16 (control strain).

Previously, we produced a yeast mutant library (>1,000 clones) that was based on *S. cerevisiae* YS59 and created by random mutagenesis (error-prone PCR) of the *SPT15* gene with the pY16 plasmid backbone using gTME technology. Preliminary screening using SD media in 24-well plates was performed to identify the 20 mutants with the highest biomass; these were the strains that were supposed to have lower ethanol-production capacity due to competition for carbon sources between biomass and ethanol production (Supplementary Figure 1). To determine the ethanol production capacity of these 20 strains, we fermented each in 10-mL of Triple M media (approximately one-fifth of the tube’s total volume; this allowed the strains to grow fully) in the tubes. Finally, the strain with the lowest ethanol yield was identified (Supplementary Table 3). Compared with the control strain, the ethanol yield of the low-ethanol-production strain (YS59-409) was significantly reduced by 34.9% (Table 1, *p* < 0.05). Interestingly, the strain featuring *SPT15* gene mutation exhibited a greater CO_2_ weight loss than the control group (Figure 1). In the wine industry, CO_2_ weight loss is generally used as an indicator of the fermentation capacity of yeast. It is worth noting that the YS59-409 strain produced a larger amount of CO_2_ than the control but had a lower ethanol production. This suggests that other decarboxylation pathways might contribute to the CO_2_ loss in the mutant strain. In addition, the glycerol content of the YS59-409 strain was 43% higher than the control strain, although there were no significant differences with regards to acetic acid production (Table 1). These findings were not consistent with the classical theory that higher glycerol synthesis is associated with increased acetic acid production (Nevoigt and Stahl, 1996). Commonly, the main by-product, glycerol, confers positive sensory effects, body, and sweetness, to wines when present in appropriate amounts and has been used as a target to redirect carbon sources away from ethanol production by many researchers in the wine industry (Tilloy et al., 2015; Goold et al., 2017). Our mutant strain (YS59-409) not only produced more glycerol than the control strain, but also did not accumulate overmuch acetic acid, an undesirable compound; these findings were also in agreement with those from previous studies (Ehsani et al., 2009; Tilloy et al., 2014). During wine fermentation, most of the sugars are used for the production of ethanol and CO_2_, with a small part for biomass and glycerol forming, including minute amounts of other byproducts. Therefore, we can speculate that strain YS59-409 uses more sugar for CO_2_, biomass, and glycerol synthesis, thus shunting the carbon source away from ethanol synthesis and resulting in low ethanol yield.

**TABLE 1:** Fermentation characteristics of the mutant strain *S. cerevisiae* YS59-409.

Strains	OD_600_	Residual sugar (g/L)	Ethanol (g/L)	Glycerol (g/L)	Acetate (g/L)	Ethanol yield^a^ (g/g Sugar)	Change in ethanol yield
YS59-pY16	4.52 ± 0.34	1.13 ± 0.35	54.67 ± 0.94	2.13 ± 0.08	0.493 ± 0.109	0.367 ± 0.007	0
YS59-409	5.45 ± 0.40*	1.90 ± 0.30	35.33 ± 3.09*	3.05 ± 0.07*	0.487 ± 0.066	0.239 ± 0.021*	−34.9%

^*a*^Ethanol yield represents the ratio of ethanol production (g) to the sugar consumption (g).* Student’s t-test showed significant difference at the level of 0.05.

### Sequence Analysis of the *SPT15* Gene in the YS59-409 Strain

**FIGURE 2:**
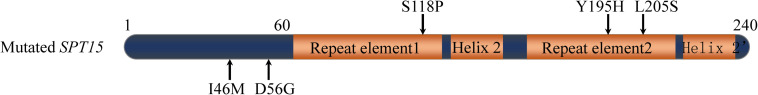
Mutation sites in the *SPT15* gene of mutant strain (arrows). The schematic of structural domain is referred to the previous study (Alper et al., 2006).

The *SPT15* gene in YS59-409 was amplified from the pY16-409 plasmid and then was sequenced. There were 5 mutational sites in the structural domain of the mutated *SPT15* gene as shown in Figure 2, in which methionine is substituted for isoleucine (Ile^46^Met), and similarly, Asp^56^Gly, Ser^118^Pro, Tyr^195^His, and Leu^205^Ser (I46M, D56G, S118P, Y195H, and L205S, respectively). Three point mutations were located in the highly conserved domain (amino acids 61–240) and two mutations were located in the non-conserved domain (amino acids 1–60). To explore which of these sites conferred the most desirable phenotype to the low-ethanol-yielding strain, we constructed five strains featuring single point mutations (Supplementary Table 1). However, none of these mutants led to a reduction in ethanol yield; instead, I46M, D56G, and L205S, led to enhanced ethanol yield compared with the control under fermentation conditions (Table 2). Of all these mutational sites, Y195H has been reported to work together with F177S and K218R to improve glucose/ethanol tolerance and the efficiency of glucose conversion to ethanol of yeast (Alper et al., 2006); and Leu-205 site has shown to be important for DNA binding specificity of Spt15p and mutating this site to other different amino acids can cause various degrees of changes in yeast growth (Arndt et al., 1992). Although our results showed that single mutation of Y195H or L205S did not produce the low-alcohol phenotype, we hypothesized that these two sites might play key roles in the mutant strain and that the desired phenotype might be obtained through double or multiple mutations based on previous reports (Arndt et al., 1992; Alper et al., 2006). In addition, the same mutation on *SPT15* may lead to different phenotypes in strains with different genetic backgrounds. In a word, these results illustrate that the combined action of the five separate mutations, or at least in part, conferred the desired phenotype to the mutant strain.

**TABLE 2:** Fermentation characteristics of point-mutated strains.

Strains	Residual sugar (g/L)	Ethanol (g/L)	Ethanol yield (g/g sugar)	Changes in ethanol yield
409-46	0.56 ± 0.91^a^	53.09 ± 0.36^b^	0.348 ± 0.005^ac^	8.10%
409-56	1.10 ± 1.67^a^	52.23 ± 1.09^b^	0.357 ± 0.018^a^	11.00%
409-118	1.04 ± 0.01^a^	51.42 ± 1.76^ab^	0.325 ± 0.005^b^	1.10%
409-195	1.03 ± 0.02^a^	50.19 ± 0.49^ab^	0.332 ± 0.003^bc^	3.10%
409-205	1.03 ± 0.02^a^	53.34 ± 3.19^b^	0.355 ± 0.013^a^	10.40%
YS59-pY16	1.02 ± 0.02^a^	48.43 ± 0.24^a^	0.322 ± 0.003^b^	0

**^*a,b,c*^Different letters indicate significant differences according to the Duncan test (p < 0.05). Data are mean ± SD of independent triplicate*.*

### Transcriptional and Metabolic Analysis of the Mutated Strain

As described in previous research, gTME can trigger overall disturbances at the transcriptional level and can be used to unravel complex phenotypes (Alper and Stephanopoulos, 2007). In order to investigate the mechanisms underlying the observed phenotype, we used RNA-Seq and metabolomic analysis to analyze the differences in transcription and metabolism between mutant strain (YS59-409) and the control (YS59-pY16). Under fermentation conditions, we observed significant changes in the transcription and metabolism of the mutant; these factors are likely to have made an important contribution to the target phenotype of the mutant. Subsequently, we would discuss the overall transcriptional and metabolic regulation and the specific pathways and function change associated with ethanol metabolism answering to the mutation of *SPT15* gene.

#### The Overall Analysis of Transcription and Metabolism in Strain YS59-409

A total of 964 genes showed significantly different expression levels when compared between the mutant and control strains; 636 genes were up-regulated and 328 genes were down-regulated in the mutant YS59-409 strain (Supplementary Table 4). These differentially expressed genes (DEGs) demonstrated that the mutation of the *SPT15* gene, a transcription factor, led to the global reprogramming of transcription at genes and may have contributed to the regulation of ethanol metabolism in this mutant strain. GO analysis and KEGG pathway enrichment analysis were used to further analyze the functional enrichment of DEGs. KEGG pathway enrichment analysis revealed that most of the DEGs participated in those key metabolic pathways, including translation, transcription, carbohydrate metabolism, nucleotide metabolism, and amino acid metabolism (Table 3).

**TABLE 3:** KEGG pathway enrichment analysis (*p*-value < 0.05).

Pathway	*P*-value	Number of genes	Gene match (genome match)^a^
**Translation**			
Ribosome	1.00E-71	135	19.77 (4.33)
**Transcription**			
RNA polymerase	5.87E-05	14	2.05 (0.69)
**Carbohydrate metabolism**			
Starch and sucrose metabolism	6.27E-10	22	3.22 (0.84)
Galactose metabolism	6.11E-04	13	1.90 (0.74)
**Nucleotide metabolism**			
Pyrimidine metabolism	1.28E-04	24	3.51 (1.65)
Purine metabolism	3.23E-02	24	3.51 (2.41)
**Amino acid metabolism**			
Histidine metabolism	4.08E-03	6	0.88 (0.26)
Arginine and proline metabolism	4.58E-02	6	0.88 (0.41)

**^*a*^The percentage of DEGs involved in individual pathway account for all DEGs with pathway annotation (683) and all genes with pathway annotation (4184).**

There were 41 differential metabolites identified (16 upregulated and 25 downregulated), as shown in Supplementary Table 6. OPLS-DA and PCA analysis were conducted to identify differences in the metabolome that were caused by random mutagenesis of the *SPT15* gene. We found there were distinct discrepancies between the mutant and control strains in terms of metabolic profile (Supplementary Figure 2). Indeed, we found that several metabolites related to amino acid metabolism (L-glutamine, L-glutamate, L-tryptophan, L-histidine, L-lysine, kynurenine, glutathione, succinic acid semialdehyde, and adenylosuccinate) (Supplementary Table 6) showed significant differences when compared between the two strains, corresponding to transcriptional alterations in amino acid metabolism.

#### Transcription, Translation, and Nucleotide Metabolism, Were Activated in Strain YS59-409

In total, 14 and 135 genes were functionally annotated into transcription and translation, respectively (Table 3 and Supplementary Tables 4, 5). Genes related to RNA polymerase were upregulated, thus indicating the transcriptional activation of the mutant strain. This could be explained by the upregulation of *SPT15* in the mutant YS59-409 strain (Supplementary Table 4) for its regulatory effect on RNA polymerase and gene transcription levels (Eisenmann et al., 1989). In addition, *RPL* and *RPS* genes encoding ribosome biogenesis proteins were significantly over-expressed in the low ethanol-production strain (Supplementary Tables 4, 5), which reflected the stimulation of relative translational activity at certain time points (Backhus et al., 2001). Coincidently, the upregulation of RNA polymerase metabolism and ribosome biogenesis were reported in a low-ethanol-production yeast by a previous study (Varela et al., 2018). Furthermore, most of the genes associated with nucleotide metabolism were expressed at high levels (Table 3 and Supplementary Tables 4, 5), of which purines and pyrimidines are the basic components (Ljungdahl and Daignan-Fornier, 2012), exhibiting the activation of purine and pyrimidine metabolism. Collectively, these results above indicate that transcription, translation, and nucleotide metabolism, were activated in the mutant YS59-409 strain. This probably demonstrates that the mutation of the *SPT15* gene leads to the reprogramming of global gene expression and metabolism.

#### The Fermentative Pathways Leading to Ethanol Production Was Reduced in Strain YS59-409

**FIGURE 3:**
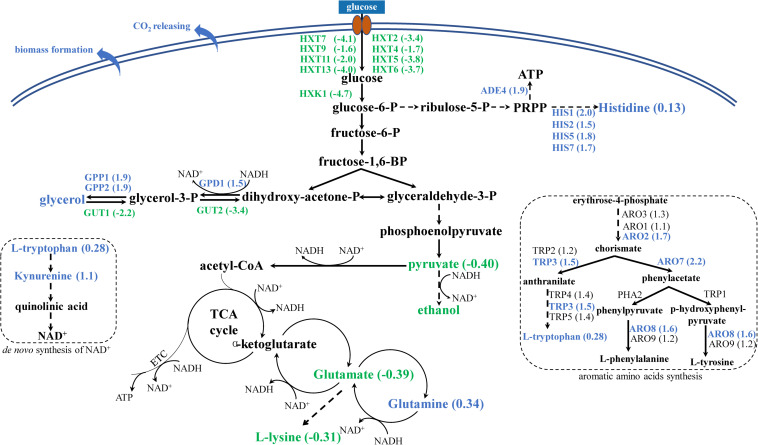
Changes in genes and metabolites of mutant strain YS59-409. Blue and green represent up- and down-regulation, respectively. The values in brackets represent the fold change of expression level of mutant strain compared to control strain.

Glycolytic flux relies on glucose uptake rate, which is regulated by a family of hexose transporters encoded by *HXT* genes. In the present study, we found that several hexose transporter genes were significantly downregulated in the YS59-409 strain (*HXT2*, *HXT4*, *HXT5*, *HXT6*, *HXT7*, *HXT9*, *HXT11*, and *HXT13*; Figure 3 and Supplementary Table 4); of these, *HXT4*, *HXT6*, and *HXT7*, are known to be vital for the uptake of glucose (Özcan and Johnston, 1999). Previous research has reported that a deficiency of hexose transporters in *S. cerevisiae* could reduce the levels of sugar uptake and thus regulate the glycolysis flux, particularly with regards to HXT7, which eventually resulted in a reduction in ethanol production (Otterstedt et al., 2004). Consequently, the significant downregulation of several *HXT* genes in YS59-409 is likely to result in a decrease of glycolysis flux. Besides, the expression of *HXK1* was downregulated by 4.7-fold (Figure 3 and Supplementary Table 4); this gene encodes hexokinase 1, which is responsible for catalyzing the phosphorylation of glucose in the first irreversible step of glycolysis (Rodríguez et al., 2001). The downregulation of *HXK1* also might contribute to the reduced glycolysis metabolism in the mutant; this observation is supported by a previous study that the repression of hexokinase activity resulted in reduced glycolysis flux (Tan et al., 2016). So, the downregulation of *HXTs* and *HXK1* in strain YS59-409 probably lead to a reduction in glycolysis flux compared to the control strain, thus well explaining the reduced yield of ethanol. Varela et al. (2018) have reported that the low ethanol strain produced in their study exhibited reduced glycolysis activity, which agrees well with the present study. What’s more, a reduction in pyruvate content (Figure 3 and Supplementary Table 6) provides further evidence of the weaker glycolysis flux (Otterstedt et al., 2004). We hypothesize that the glycolysis flux of the YS59-409 strain is reduced by mutation of the *SPT15* gene, thus resulting in a weakened ethanol fermentation pathway of the YS59-409 strain.

#### The Low-Ethanol-Producing Strain YS59-409 Exhibited a Disturbance in the Crabtree Effect

*Saccharomyces cerevisiae* is a Crabtree-positive yeast, which generally utilizes the ethanol fermentation pathway for glucose consumption under conditions of excess glucose, even in the presence of oxygen (also referred to as ‘overflow metabolism’) (de Deken, 1966). In the present study, we observed indications that the Crabtree effect of the YS59-409 strain might be disturbed. On the one hand, the attenuated glycolysis flux in the YS59-409 strain, in combination with the higher CO_2_ production and the lower ethanol formation rate, appeared to suggest that the TCA cycle was enhanced. This deduction could be supported by an earlier study showing that when glucose uptake rates were reduced, then the CO_2_/ethanol ratio increased more than 50% and the net flux through the TCA cycle increased significantly (Heyland et al., 2009). That is to say, the overflow metabolism of the YS59-409 strain might shift toward respiratory metabolism. Our specific experimental conditions (the headspace of the 24-well plates and test tubes) could provide a micro-oxygen environment that can support the respiration of yeast to some extent, as also noted by a previous study (Heyland et al., 2009). Respiratory metabolism plays an important role in terms of producing energy in the form of ATP in aerobic growing cells. Intriguingly, we found that the energetic metabolism of YS59-409 may be enhanced compared to the control strain. As is well-known, the histidine and nucleotide biosynthetic pathways are connected (Ljungdahl and Daignan-Fornier, 2012). The upregulation of genes related to histidine metabolism (such as *HIS1*, *HIS2*, *HIS5*, and *HIS7*), accompanied by the increased histidine content in YS59-409 (Figure 3), provided strong evidence for an enhancement in the synthesis of histidine. And KEGG pathway enrichment analysis indicated that purine synthesis metabolism was reinforced in the mutant strain (Table 3 and Supplementary Tables 4, 5). So, with the viewpoint that the *de novo* purine pathway feeds into the histidine pathway and branches to allow ATP synthesis (Ljungdahl and Daignan-Fornier, 2012), we can speculate that ATP production in the YS59-409 strain is boosted. In addition, the upregulation of the *ADE4* gene in the first step of the ATP formation pathway (Ljungdahl and Daignan-Fornier, 2012) provided supplementary evidence to the enhanced ATP synthesis. What’s more, high ATP yields may result in excess biomass formation at the expense of product yield (de Kok et al., 2012), which further supports the fact that YS59-409 showed higher biomasses and lower ethanol production. Therefore, we can infer that the characteristic of Crabtree effect of the mutant strain have been changed. On the other hand, some Crabtree-negative yeast, such as *Hanseniaspora uvarum* and *Metschnikowia pulcherrima*, possess different respiro-fermentative regulatory mechanisms than *S. cerevisiae* (Quiros et al., 2014). Researchers have used these non-conventional species to reduce ethanol production by partial controlling the aeration of grape juice (Quiros et al., 2014). However, non-conventional yeasts cannot generally complete alcoholic fermentation. We created a new mutant strain (YS59-409), which might not only have similar ethanol-producing properties as non-conventional yeasts, but also is capable of finishing fermentation alone. Thus, these results provide a new concept for the creation of new low-ethanol-production strains.

#### NAD^+^/NADH Homeostasis Was Disturbed in Strain YS59-409

Sugar fermentation in *S. cerevisiae* is a redox neutral process that is influenced by NAD^+^/NADH balance, in which glycerol plays important roles (Goold et al., 2017). We found those key genes in the synthesis pathway of glycerol, *GPD1*, encoding glycerol-3-phosphate dehydrogenase, along with *GPP1* and *GPP2*, encoding glycerol-3-phosphate phosphatase (Nevoigt and Stahl, 1997), were overexpressed in the low-ethanol-producing strain (Figure 3 and Supplementary Table 4). Simultaneously, *GUT1* and *GUT2*, genes that encode for glycerol kinase for glycerol catabolism (Nevoigt and Stahl, 1997) were both downregulated (Figure 3 and Supplementary Table 4). Changes in the expression of those genes related to glycerol metabolism demonstrated the high production of glycerol; this was confirmed by the increased glycerol concentration in the YS59-409 strain (Table 1). Higher production of glycerol is likely due to the need to balance cytosolic NADH produced and consumed. What is noticeable is that the *de novo* pathway of NAD^+^ (namely, the kynurenine pathway) might have been disturbed. In yeast, NAD^+^ can be synthesized *de novo* from tryptophan (Panozzoa et al., 2002). We found that tryptophan content was increased in the mutant strain (Figure 3 and Supplementary Table 6); this was consistent with the fact that genes involved in the synthesis of tryptophan were also increased (Figure 3 and Supplementary Table 4). Moreover, as the key participator in the *de novo* synthesis of NAD^+^ from L-tryptophan (Panozzoa et al., 2002), kynurenine was found to be the increased metabolite with the highest fold-change in this study (Figure 3 and Supplementary Table 6). Previous studies have shown that the *de novo* pathway plays only a minor role if a functional salvage pathway is present (Sporty et al., 2009); and one of the key requirements in the formation of kynurenine is that the kynurenine pathway needs oxygen (Panozzoa et al., 2002). Therefore, the increased production of kynurenine and L-tryptophan in the mutant strain probably shows the activation of the *de novo* pathway for the synthesis of NAD^+^ under our experimental conditions. The strengthening trend of NAD^+^ level might further explain the enhanced synthesis of ATP synthesis for the reason that ATP synthesis and redox potential are directly proportional to the intracellular concentration of NAD^+^ (Gonzalez Esquivel et al., 2017). Collectively, those data indicate that NAD^+^/NADH equilibrium might be disturbed in the mutant strain. Certainly, the NAD^+^/NADH balance of the YS59-409 strain depends on a range of factors, including biomass formation, respiration, ATP production, and the generation of some metabolites, such as ethanol, glycerol, and amino acids. Additionally, researchers have deployed methods to intentionally perturb the NAD^+^/NADH balance to reduce ethanol production (Goold et al., 2017), which also emphasizes the importance of NAD^+^/NADH balance for ethanol metabolism.

### The Effects of Deleting the *RGI1/2* Gene on the Ethanol-Production Capacity of Yeast

**FIGURE 4:**
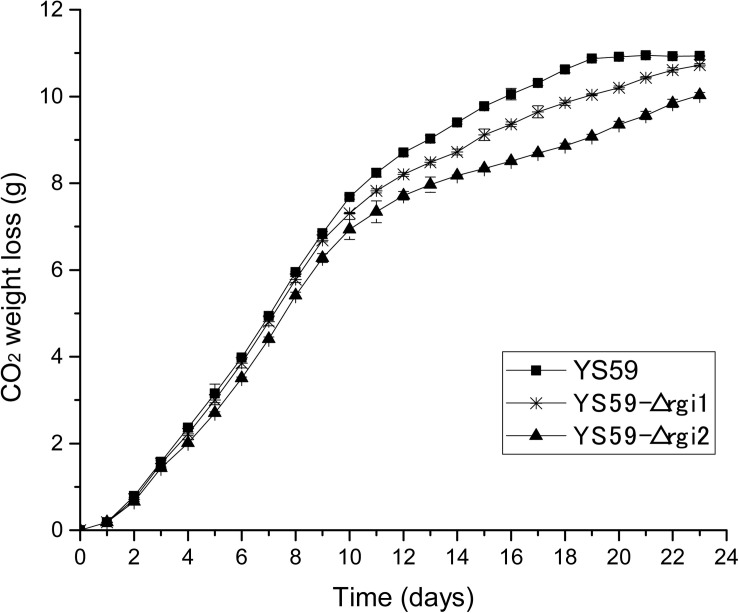
CO_2_ weight loss (g). Black asterisk and black triangle represent knockout strains (YS59-Δrgi1 and YS59-Δrgi2, respectively) and black square represents control strain (YS59).

To further identify key genes in the regulatory network of our new mutant strain, we constructed a protein-protein interaction (PPI) network using the STRING database V11^3^ and Cytoscape tools; the core gene module was then excavated using the MCODE plugin in Cytoscape. Nine genes were identified (Supplementary Table 7); of these, *AQY3* (Yfl054c) and *RGI2* (Yil057c) were shown to be associated with glycerol and energy metabolism, which was corresponded with the changes in glycerol and energy metabolism observed in YS59-409. Then, we found that only *RGI2* exerted influence over ethanol production. The *RGI2* gene possesses very little data concerning protein function or biological processes involved for itself, which was found to be significantly down-regulated by 41.9-fold in this study (Supplementary Table 4). Noteworthily, the homologous gene of *RGI2*, *RGI1* (Yer067w), was significantly down-regulated by 4.3-fold (Supplementary Table 4), which shared 70% identity with the sequence of *RGI2* (Domitrovic et al., 2010). *RGI1* gene is reported to be regulated transcriptionally by *SPT15* under conditions involving ethanol stress (Yang et al., 2011). Previous studies show that Rgi1p and Rgi2p proteins most likely belong to the same complex and/or operate in the same pathway, and that these proteins are involved in the control of energetic metabolism, particularly under respiratory growth conditions (Domitrovic et al., 2010). Therefore, the effects of those two genes on ethanol metabolism were conducted in the present study. We performed single gene knock-outs for *RGI1* and *RGI2* genes in strain YS59 using Cre/loxP-mediated technology in order to evaluate their effects on ethanol production in *S. cerevisiae*, generating two new strains: YS59-Δrgi1 and YS59-Δrgi2 (Supplementary Table 1). As follow, we compared the performance of strain YS59-Δrgi1 and strain YS59-Δrgi2 in Triple M media (250-mL flasks containing 150-mL of media). Compared with the control strain YS59, the weight loss of CO_2_ was both reduced in the two knockout strains (Figure 4), thus illustrating their reduced fermentation capacity; this was further supported by the fact that these strains showed a 22.0 and 16.5% reduction in ethanol productivities, respectively (Table 4). The results implied that perturbation of the *RGI1* or *RGI2* gene could elicit alterations in ethanol metabolism, which showed the positive effects of *RGI1/2* deletion with regards to weakening the ethanol-yielding ability of yeast. However, these two knockout strains may have a different low-yield ethanol mechanism than the YS59-409 strain, because they had different CO_2_ production models; this possibility requires further investigation.

**TABLE 4:** Fermentation characteristics of knockout strains.

Strains	Residual sugar (g/L)	Ethanol (g/L)	Ethanol yield (g/g Sugar)	Change in ethanol yield
YS59	1.96 ± 0.68^a^	52.04 ± 1.43^c^	0.351 ± 0.008^c^	0
YS59-Δrgi1	0.99 ± 0.19^a^	40.59 ± 0.96^a^	0.272 ± 0.006^a^	−22.0%
YS59-Δrgi2	1.62 ± 0.40^a^	43.43 ± 0.77^b^	0.292 ± 0.004^b^	−16.5%

**^*a,b,c*^Values followed by different letters within the same column are significantly different using Duncan’s multiple range test at the level of 0.05. Data are mean ± SD of independent triplicate.**

## Conclusion

In this study, we applied gTME technology to engineer a low-ethanol-production strain of *S. cerevisiae* achieved by mutating the transcription factor *SPT15* to change gene expression in a global level. We provide a novel insight into the use of gTME technology to modulate ethanol metabolism, which could not only facilitate the construction of a low-ethanol-production strain for the wine industry, but also, enhance our understanding of the mechanisms underlying reduced ethanol production by yeast. We created a new strain of *S. cerevisiae*, YS59-409, with weakened ethanol production capacity. The ethanol-production capacity of this strain was reduced by 34.9% compared to the control strain, which was caused by comprehensive changes associated with the regulation of transcription and metabolism. Sequence analysis was performed on the mutated *SPT15* gene, demonstrating that the five mutation sites may work collectively, or at least partly, to create the specific characteristics of YS59-409, including a higher CO_2_ release, biomass, and glycerol formation. The integration of RNA-Seq and metabolomics analysis showed that the specific phenotype of the new mutant strain featured changes in ribosome biogenesis, nucleotide metabolism, glycolysis flux, the Crabtree effect, NAD^+^/NADH homeostasis, and energy metabolism. Remarkably, we also found that *RGI1* and *RGI2* genes, which play key roles in energy metabolism, were significantly down-regulated; it is possible that this was linked with low ethanol metabolism, although this needs to be investigated further in future research. Although public attitudes toward the use of GMOs (Genetically Modified Organisms) in wines are often less than positive, this study demonstrated that it is possible to reduce ethanol yields in yeast using gTME technology, while also reprogramming the metabolism of this new mutant strain. Currently, we can get some knowledge from this study, which could be used to direct strategies for generating wine yeast with weakened ethanol production capacity using other approaches, such as adaptive evolution. In summary, this study highlights the potential to use gTME technology to reduce the ethanol content of yeast for the wine-making industry.

## Data Availability Statement

The datasets generated for this study can be found in online repositories. The names of the repository/repositories and accession number(s) can be found in the article/Supplementary Material.

## Author Contributions

QD: experimental operation and writing – original draft. YL: supervision. YS: data analysis. YQ: experimental design and supervision. All authors edited and approved the final version of the manuscript.

## Conflict of Interest

The authors declare that the research was conducted in the absence of any commercial or financial relationships that could be construed as a potential conflict of interest.

## Footnotes

1 http://metlin.scripps.edu

2 http://www.metaboanalyst.ca

3 https://string-db.org/
