# Supplementary material for: Creation of a Low-Alcohol-Production Yeast by a Mutated SPT15 Transcription Regulator Triggers Transcriptional and Metabolic Changes During Wine Fermentation
Source: Front Microbiol. 2020 Dec 14;11:597828. doi: 10.3389/fmicb.2020.597828 (PMC7768003; doi:10.3389/fmicb.2020.597828)
Supplement: Supplementary file 1 [file Data_Sheet_1.PDF]

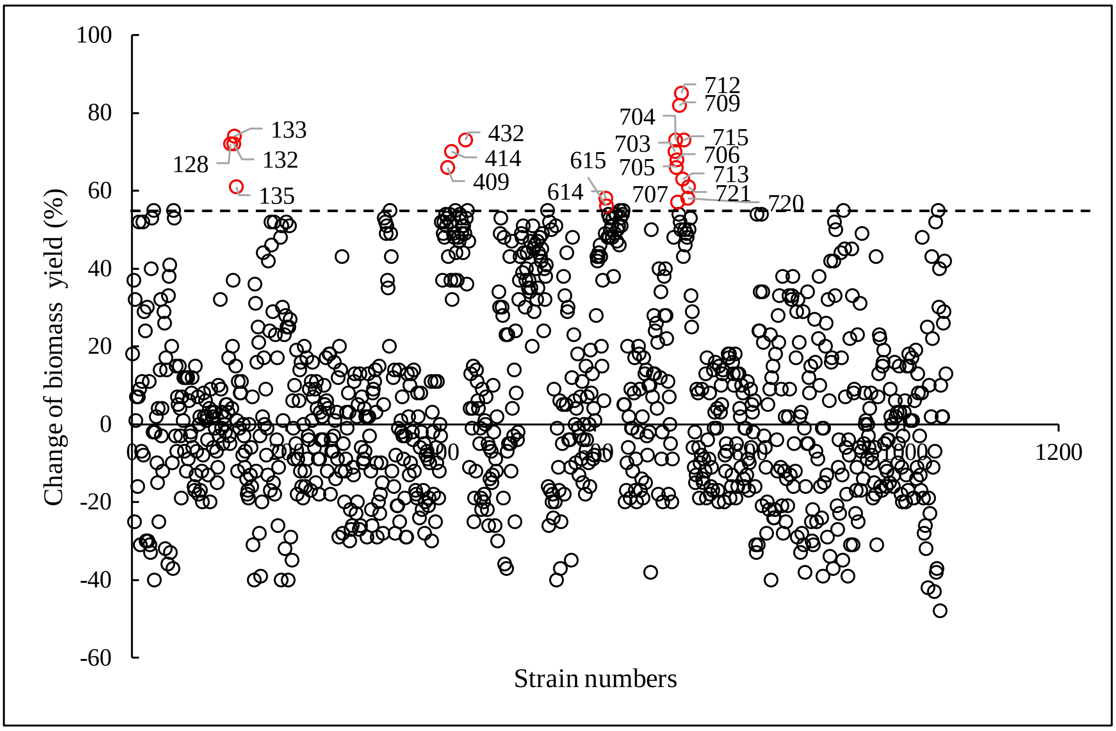

FIGURE S1. Preliminary screening of the yeast mutant library. Biomass yield (g/g sugar) represents the ratio of biomass formation (g) to the consumption of sugar (g). Change of biomass yield (%) represents the ratio of biomass yield change compared with control strain. A total of 1053 transformants were cultivated using SD media in 24-well plates and 20 mutants with the highest biomass yield change were labelled with strain numbers and marked in red.

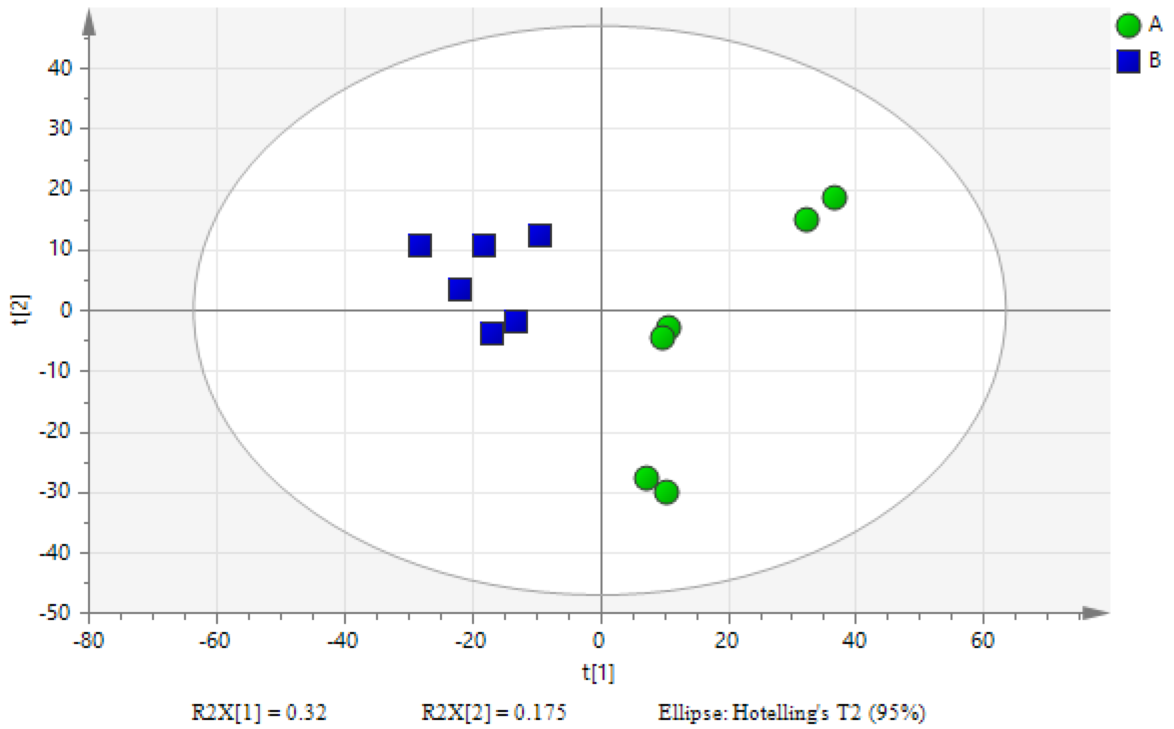

(a)

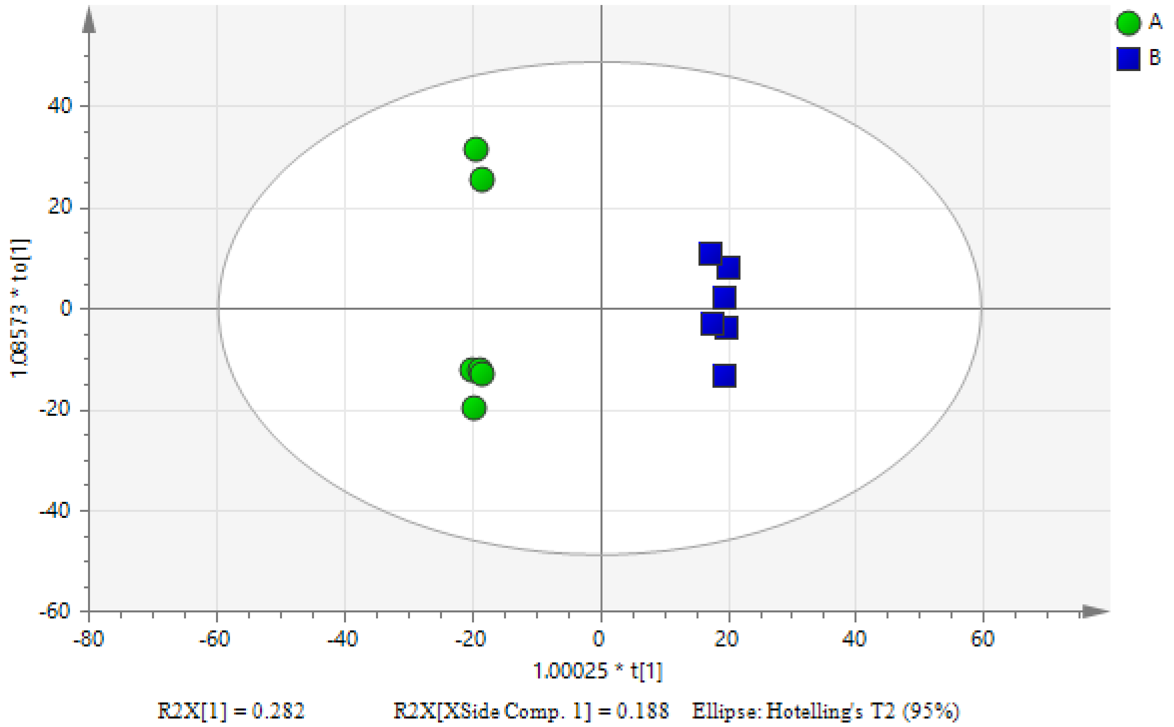

(b)

FIGURE S2. Scores plot of PCA (a) and OPLS-DA (b). A represents YS59-pY16 (control strain) and B represents YS59-409 (low-ethanol-production strain).
